# Analysis of polychlorinated biphenyls (PCBs) in edible oils using the QuEChERS/GC‐MS method: A health risk assessment study

**DOI:** 10.1016/j.heliyon.2023.e21317

**Published:** 2023-10-24

**Authors:** Hadi Eghbaljoo, Mohammad Rezvani Ghalhari, Nabi Shariatifar, Gholamreza Jahed Khaniki, Ebrahim Molaee Aghaee, Mahmood Alizadeh Sani, Elahe Mansouri, Majid Arabameri

**Affiliations:** a Division of Food Safety and Hygiene, Department of Environmental Health Engineering, School of Public Health, Tehran University of Medical Sciences, Tehran, Iran; b Department of Environmental Health Engineering, School of Public Health, Tehran University of Medical Sciences, Tehran, Iran; c Department of Clinical Nutrition, Faculty of Nutritional Sciences and Dietetics, Tehran University of Medical Sciences, Tehran, Iran; d Food Safety Research Center (salt), Semnan University of Medical Sciences, Semnan, Iran

**Keywords:** Polychlorinated biphenyls, Health risk assessment, Edible oils, Gas-chromatography mass spectrophotometry (GC/MS)

## Abstract

Population growth has made it difficult to provide safe food; because various toxic substances such as polychlorinated biphenyls (PCBs) can contaminate food products such as edible oils which have very high-rate consumption worldwide. Aims of study are to determine the concentration of PCBs in edible oil samples and to evaluate the carcinogenic risk of PCBs in them among Iranian people by Monte Calo Simulation (MCS). After finding the location of high customer hyper market in Tehran, 42 samples of 7 various types of edible oils were collected; then PCBs content of them measured using the modified QuEChERS (Quick, Easy, Cheap, Effective, Rugged, and Safe) extraction method and GC/MS technique. According to the findings the mean level of NDL-PCB in oil samples were ranged from 1.88 to 25.62 ng/g fat. Results of uncertainty analysis showed that among children the 95th percentile of Incremental Lifetime Cancer Risk (ILCR) were 7.80E-3, 5.37E-4, 5.37E-4, 2.00E-3, 1.59E-3, 13.9E-3 and 7.04E-4 for animal oil, corn oil, frying oil, olive oil, bran oil, sesame oil and sunflower oil, respectively. Also, the 95th percentile of ILCR among adults were 4.12E-2, 3.04E-3, 3.09E-3, 1.06E-2, 8.43E-3, 7.38E-3 and 3.74E-3 for animal oil, corn oil, frying oil, olive oil, bran oil, sesame oil and sunflower oil, respectively. The risk evaluation showed that in all edible oils the 95th percentile of simulated ILCR were more than 10^−5^, so it can be threatening health among both aging groups, although, the producers don't deliberately subject the users' lives to such danger, but high consumption rate and accumulation of PCBs in body tissues are contribute to increasing carcinogenic risk. Also, the 95th percentile of ILCR among adults were more than children, because the edible oil ingestion rate among adults was more than children.

## 1 Introduction

One of the indispensable parts of diet that used in cooking are edible oils, and their per capita consumption is very high worldwide [1]. Edible oils have many types based on their applications; among edible oils, vegetable oils and some animal oils are used much more than others in the cooking process and food industry. Edible oils can be one of the primary sources of human exposure to contaminants and toxic substances [2,3]. various toxic substances can contaminate food products, including mycotoxins, heavy metals (HMs), pesticides, polychlorinated biphenyls (PCBs) and Polycyclic aromatic hydrocarbons (PAHs) [4].

The PCBs are the main these contaminants with the chemical formula C12H10-nCln are divided into non-dioxin-like (NDL)-PCBs (including PCBs 28, 52, 101, 138, 153, and 180) and dioxin-like PCBs [5]. PCBs are most frequently found in foods including meat, dairy products, seafood, eggs, and edible oils. The relatively low quantity of these pollutants in edible oils is also a problem because they make up a significant portion of the human diet [6,7]. PCBs can enter the human body through food, water and air and accumulate in fatty tissues. These contaminants are found in a wide range of biological samples due to their accumulative properties, high stability, hydrophobicity, low volatility, and high resistance to metabolic degradation, with a half-life of about 7–10 years [8,9]. Based on the European Union (EU) guidelines in the vegetable and animal oils the maximum level of NDL-PCBs is 40 ng/g (fat wt) [10]. It has been proven that over 90 % exposure to PCBs is through the food and can lead to endocrine, immunological disorders, and reproductive disorders [11,12]. Also, according to International Agency for Cancer Research (IACR) reports the PCBs are carcinogen [13]. Therefore, due to the hydrophobic nature of PCBs, the possibility of their presence in fat/oil samples is very high.

Several tools and methods have been created over the last twenty years to find NDL-PCBs in food. However, gas chromatography (GC) is still the most popular and accurate way to find these chemicals. The use of a gas chromatograph equipped with an electron capture detector (ECD) or mass spectrometry (MS) is the most technologically sophisticated and accurate method for locating PCBs in fats, oils, and other types of foods. Because the monitors are very sensitive to PCBs and other substances that contain chlorine, this method can have a low specific limit of detection (LOD) [[14], [15], [16]].

Compared to traditional extraction methods; recently, the QuEChERS (Quick, Easy, Cheap, Effective, Rugged, and Safe) protocol used for the analysis and detection of various contaminants and residues in different food matrices such as fruits,vegetables, meats, seafood, oils, dairy products, etc. [[17], [18], [19], [20], [21]]. Many researches have confirmed this protocol to detect the pesticides [18], polycyclic aromatic hydrocarbons (PAHs) [22] matrices [23], food additives [24], and heterocyclic amines in foodstuffs [25]. , the QuEChERS protocol is an efficient, applicable and the most reliable analytical procedure in foodstuffs analysis.

Edible oils have a high-rate consumption among Iranian households, there is no comprehensive study for PCBs detection among various types of edible oils worldwide especially Iran. Therefore, the present study aimed to:1) investigate the concentration of NDL-PCBs in edible oil samples using the modified QuEChERS extraction method and GC/MS technique and 2) assess the carcinogenic risk of PCBs in various types of high-rate edible oils among Iranian people by Monte Calo simulation (MCS).

## 2 Experimental

### 2.1 Sample collection

First of all, by the Google Earth all hyper markets of Tehran to collect edible oils were determined. Then, 42 samples of 7 various types of edible oils (sunflower oil (n = 6), corn oil (n = 6), olive oil (n = 6), sesame oil (n = 6), rice bran oil (n = 6), frying oil (n = 6), and animal oil (fat) (n = 6)) were collected from high customer hyper market. Then, edible oil samples were transported to the laboratory and stored at 4 °C before preparation and analysis.

### 2.2 Sample preparation and analysis

Before starting the preparation and analysis process, to reduce errors, all containers in the food chemistry lab were carefully rinsed with water, then all samples were immersed in nitric acid for 24 h, and finally, all sampled were washed by distilled water. in the next step, 5 mL of acetonitrile (CH_3_CN) was added to 5 g of the homogenized edible oil, and then 0.05 g of magnesium sulfate was added to this solution. then, samples were completely mixed by a shaker for 5 min and then placed in an ultrasonic homogenizer for 10 min at 40 °C. Afterward, samples were centrifuged at 5000 rpm for 3 min. In the following, 6 mL of the supernatant phase was transferred into a glass tube and 50 μL of the internal standard (PCB-29) solution was added to the glass tubes and stirred to determine the recovery percentage. In order to defatting, the samples were placed in the freezer (−18 °C for 2 h), and then the frozen fats were separated by filter paper No. 42. then, 0.25 g of primary secondary amine (as solid phase extraction) was added to the resulting material for deproteinization and stirred for 30 s and mixed with a shaker for 5 min. In the next step, the tubes containing the samples were centrifuged at 5000 rpm for 3 min. The resulting material was transferred into a container and mixed with 0.5 mL of 5 % formic acid to acidify.

1 μL of the sample final was injected into the GC-MS to analyze and measure PCBs. Samples were analyzed to detect 6 PCBs types including PCBs 28, 52, 101, 138, 153, and 180.

### 2.3 GC–MS instrument and condition

The GC/MS system used for the analysis and determination of PCBs was the Agilent 7890A, with a Mass Selective detector: 5975C VL MSD with Triple-Axis detector, from the USA. The following conditions that mentioned in Table 1 were employed for the analysis.

**Table 1:** The GC–MS instrument and condition that used to analysis of NDL-PCB in edible oils.

Parameter	Value	Unit
Sample volume	1	μL
Initial temperature	40	°C
Initial time	1	min
Ion source temperature	230	°C
Injection port temperature	230	°C
Ion source: Electron impact (EI)	0	eV
Analyzer	Quadrupole	
Flow rate	1	mL/min
Final time	10	min
Split rate	100	mL/min
Final temperature	270	°C
Program rate	3	°C/min
Carrier gas	Helium 99.1 %	

Column: Rtx 5 MS (Length: 30 m, I.D.: 0.250 mm, Film thickness: 25 μm).

### 2.4 Health risk assessment (HRA)

Risk assessment demonstrates the carcinogenic and non-carcinogenic effects on human based on the nature of pollutants that mentioned in the literature. Based on Integrated Risk Information System (IRIS) the PCBs just have carcinogenic effect on human; so, in present study, carcinogenic effect of NDL-PCBs in edible oils among adults and children was investigated. HRA has four steps including: (1) Hazard identification, (2) exposure assessment, (3) toxicity assessment, and (4) risk characterization, which these steps should be carried out respectively and precise. To calculating and evaluating chemical agent such as PCBs risk, United States Environmental Protection Agency (US-EPA) developed a mathematical method that can estimate health risk due to exposure to various concentration of PCBs, one of them which can assess carcinogenic health risk by using the chronic daily intake (CDI) (mg/kg-day) concept as Eq. (1).

(Eq. 1) $$\text{CDI}=\frac{C\times \text{IR}\times \text{EF}\times \text{ED}}{\text{BW}\times \text{AT}\times 1000}$$

where C is the NDL-PCBs (ng/g), IR is the edible oil ingestion rate (g/day), the exposure frequency to PCBs considered for a full year (365 days/year) which defined by EF, ED is the exposure duration to PCBs during the life time (Years), BW is the average body weight of the edible oils consumers in Tehran, and AT is the average time for exposure to PCBs among Iranian children and adults (ED × 365 days). All parameters for HRA evaluating are shown in Table 2, and NDL-PCBs concentration is mentioned in Table 3.

**Table 2:** Values of parameters used in health risk assessment of PCBs in edible oil among children and adults.

Parameters	Unit	Symbol	Children	Adults	Reference
PMs	ng/g	C	Table 3	Table 3	Results
Ingestion Rate	g/day	IR	20	45	[26]
Exposure Frequency	Days/Year	EF	365	365	[27]
Exposure Duration	Year	ED	6	40	[27]
Body Weight	Kg	BW	15	72	[28]
Average Time (for cancer)	Days	AT	25550	25550	[27]
PCBs cancer slope factor	mg/Kg	CSF	2	2	[29]

**Table 3:** Mean PCBs in edible oils samples collected from Iran (ng/g fat).

oil type		PCB 28	PCB 52	PCB 101	PCB 138	PCB 153	PCB 180	Total NDL-PCB
sesame	Mean	ND	1.24	1.95	ND	1.42	ND	4.61
	SD	–	0.09	0.03	–	0.10	–	0.07
animal oil	Mean	ND	10.71	14.75	ND	0.17	ND	25.62
	SD	–	1.03	1.00	–	0.01	–	1.01
corn	Mean	ND	1.29	0.42	ND	0.16	ND	1.88
	SD	–	0.93	0.03	–	0.02	–	0.09
frying	Mean	ND	0.35	0.38	ND	1.20	ND	1.93
	SD	–	0.01	0.06	–	0.02	–	0.02
olive	Mean	ND	1.25	5.08	ND	0.31	ND	6.63
	SD	–	0.01	0.17	–	0.01	–	0.01
rice bran	Mean	ND	3.92	0.38	ND	0.90	ND	5.19
	SD	–	0.02	0.06	–	0.04	–	0.30
sunflower	Mean	ND	0.31	0.34	ND	1.64	ND	2.29
	SD	–	0.02	0.05	–	0.20	–	0.15
Total	Mean	ND	2.40	2.64	ND	0.86	ND	5.91
	SD	–	0.10	0.2	–	0.01	–	0.18

ND: Not detection.

The Incremental Lifetime Cancer Risk (ILCR) from the US-EPA was used in this study to figure out how toxic PCBs in food oils are. The carcinogenic effect of NDL-PCBs in the edible oils were calculated using Eq.2:

(Eq 2) ILCR=CDI×CSF

After calculating ILCR, the amount of likely cancer risk should be in line with World Health Organization (WHO) targets. If the upper bond of simulated risk (95th percentile) was less than 10E-06, it means that there is not an excessive cancer risk from NDL-PCBs in edible oils. If the 95th percentile of ILCR was between 10E-04 and 10E-06, it means that low priority was needed and more research was needed before either taking action or not.

### 2.5 Statistical analysis

Data were analyzed using SPSS 21 software (Chicago, IL, USA). The reported data includes the mean, standard deviation, minimum, and maximum. The normality of the data distribution was assessed using the Kolmogorov-Smirnov analysis. In present study, the CDI and ILCR of NDL-PCB in edible oils were calculated using MCS by Crystal Ball (Crystal Ball v 11.1.2.4.600 software, Oracle, Redwood City, CA, USA). The MCS method, introduced by USEPA, is an improved approach to communication with uncertainty for risk management, ensuring food safety.

## 3 Results and discussion

In this study, six important NDL-PCBs in the food chain were analyzed in 42 samples of edible oils. Table 3 presented the mean total of NDL-PCBs in the samples was 5.91 ng/g (maximum: 25.62 ng/g and minimum: 1.88 ng/g). Among measured PCBs, PCB-101 had the highest concentration (14.75 ng/g, in edible animal oils), while the lowest PCB content was associated with PCB-153 (0.16 ng/g, in corn oils) (Table 3). Also, the LOD (0.12–0.32), LOQ (0.6–1.7), R% and RSD for all PCBs detected in edible oils were presented in Table 3.

The level of total NDL-PCB of animal oils (25.62 ng/g fat) was lower than the European Food Safety Authority (EFSA) standard. In this regard, these concentration of the NDL-PCB in animal oils may be due to water pollution, air pollution, pollution during the oil extraction process, atmospheric factors, animal feed (about animal oils), and the proximity of farms to centers industrial [30]. NDL-PCB levels are higher in animal oils than in vegetables oils for these reasons. Conversely, compared to other vegetables oils, olive oil had the most PCB. Pesticides are possibly the main reason why olive oil is so polluted compared to other vegetable oils [31].

The content of 11 PCBs in different food products was assessed by Llobet et al. (2003). The findings of the study revealed that fish and oysters had the greatest concentration of PCBs, with levels reaching 11864.18 ng/kg. The presence of pollutants in fish and oyster samples can be ascribed to the unauthorized discharge of industrial waste into marine environments. Moreover, the elevated concentration of fat in fish might potentially be a contributing factor to the observed contamination levels. After fish and oysters, milk and dairy products had the second highest concentration of PCBs at a level of 674.5 ng/kg fat [32]. Baars et al. (2004) did a study where they looked at the amounts of PCDDs, DL-PCBs, and NDL-PCBs in different kinds of food. When they looked at vegetable oils, they found that they had 0.18 pg/g of fat PCDD/Fs + DL-PCBs and 1.3 ng/g of fat index PCBs which is lower than EU guidelines which is same as present study findings [33]. The amounts of PCBs and chemicals were checked in olive oil samples that were still whole by Yague et al. (2005). When the experts looked at one of the samples, they found that it had 10 μg/kg of only one organophosphate pesticide. 20–90 % of the samples had PCBs found in them, at amounts range from 2.3 to 17.3 μg/kg fat. In 5–47 % of the samples, low amounts of organochlorine herbicides were also found. A lot of chemicals may have gotten into these oils because they were used on olive trees without thinking. It is interesting to note that the study's results match up with our own. Also, the PCB amounts that were looked at were less than the EU limit of 40 ng/g fat [31].

Malavia et al. (2007) analyzed the concentrations of PCDDs, PCDFs, and DL-PCBs in six different varieties of maize oil, the information revealed that some samples of corn oil contained more of these contaminants than was permitted. This discrepancy demonstrates how dissimilar their outcomes are from those of our research [6]. In a different study, Leondiadis et al. (2008) looked at different food samples to see if they contained dioxins and DL-PCBs. It's interesting that the amounts of these pollutants in all the food samples that were tested were less than what the EU allows. This matching of results adds to the strength of our conclusions, especially when it comes to olive oil [34]. Adenugba et al. (2008) say that they measured the amounts of PCBs in food oils and goods that contain oil. It was found that the total amount of PCB in food oils was between 4.73 and 44.38 ng/g. The range for mayonnaise was 1.40–6.18 ng/g, for seafood sauce it was 1.28–5.64 ng/g, and for salad cream it was 1.21–6.25 ng/g fat. It is possible for pollutants to move from the air to water and land. Several things affect the spread of poisoning, including the speed and direction of the wind, the chemical stability of PCBs, air pressure, humidity, temperature, and the state of the environment. In general, this study's results agree with ours in that the amount of these pollutants is less than the allowed limit [35].

Dine et al. (2015) looked into how much organochlorine herbicides, their byproducts, and PCBs were in olive oil samples. There were between 4.2 and 48.7 μg/L of organochlorine herbicides in the olive oil samples; also, early 5.2 ng/g fat were found in the olive oil samples as a whole. For this study, which was like the one we did, all of the samples had PCB amounts below the highest limit. PCBs can be found in edible oil samples because of many things, including industry methods used to make edible oil, land pollution, atmospheric factors, and more [36]. Another study by Pemberthy et al., in 2016 examined the concentrations of PCDDs, PCDFs, and DL-PCBs in several market foods, including olive oil, soybean oil, fish oil, butter, and shrimp. The highest levels of PCDD/Fs and DL-PCBs were predicted to be found in fish oils and shrimp. For the same reason, PCDD/Fs and DL-PCB levels were elevated in samples of butter and soybean oil. Of all the dietary oils examined in this study, olive oil had the fewest contaminants [37]. Fathabad et al. (2019) conducted a study about PCBs content of Fish samples collected from the Caspian Sea; cyprinus carpio, a type of fish, had the highest concentration of DL-PCBs (1156.27 14.55 pg/g fat), whereas Vimba had the lowest concentration (232.43 16.05 pg/g fat). The amount of DL-PCB in certain fish can vary depending on factors such their fat content, where they come from, the weather, and the season [9]. In a study by Shahsavari et al. the amount of six NDL-PCBs in ice cream and other cream products was determined using gas chromatography (GC) and the QuEChERS technique. The average total content of NDL-PCBs in cream samples (21.634 2.18 ng/g fat) was found to be higher than in ice cream samples (12.317 1.524 ng/g fat). This distinction suggests that these pollutants possess a property that attracts fat [38].

### 3.1 Probabilistic health risk assessment

The results indicate that the average dietary intake of NDL-PCBs found by oil consumption was 2.45 ng kg^−1^ BW for adults and 5.28 ng kg^−1^ BW for children, as shown in Table 4. The mean CDI value of six NDL-PCBs congeners across all samples was found to be below the World Health Organization's recommended guideline value for food production (CDI <10 ng kg^−1^ BW.day^−1^) [8].

**Table 4:** Method validation parameters.

PCB type	LOD	LOQ	%RSD	%Recovery
PCB-180	0.32	1.07	9.50	99.3
PCB-153	0.32	1.07	8.01	100.2
PCB-138	0.12	0.41	7.27	103.1
PCB-101	0.14	0.47	6.11	98.6
PCB-52	0.21	0.70	5.60	102.4

Kang et al. (2012) found that the estimated daily intake (EDI) of indicator PCBs from food in China was 26.47 ng/kg greater than the EDI obtained in our study [39]. Chung et al. (2018) found that the minimum and upper bounds of exposure to indicator PCBs in Hong Kong were 0.68 and 1.38 ng/kg day, respectively [40]. Several European investigations have shown that the exposure to indicator PCBs in France, Italy Netherlands were 7.7 [41], 10.9 [42], and 5.6 ng/kg day, respectively [33].

**Fig. 1:**
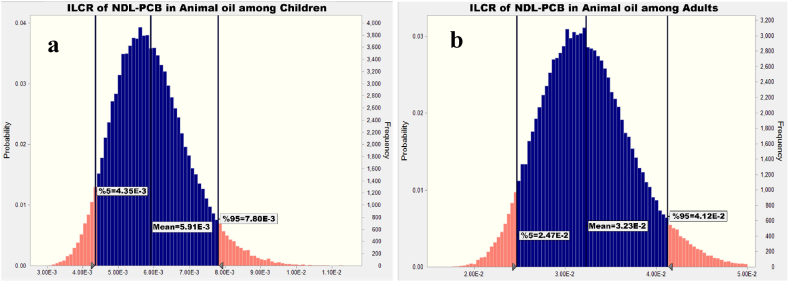
ILCR assessment of NDL-PCB in animal oil among (a) children and (b) adults.

**Fig. 2:**
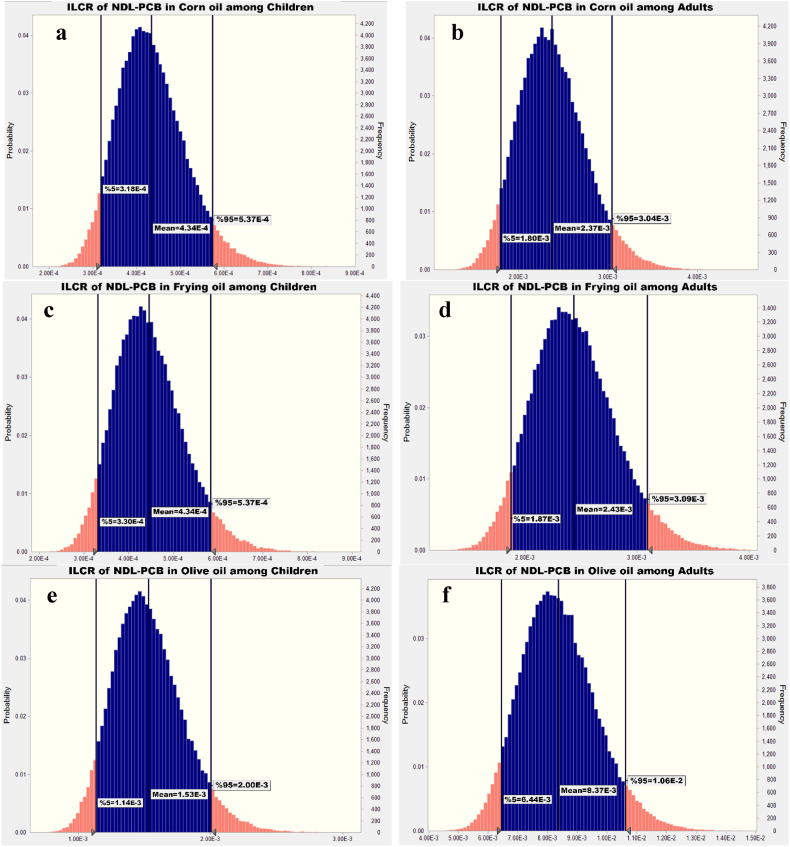
ILCR assessment of NDL-PCB in corn oil (a,b), frying oil (c,d) and olive oil (e,f) among children and adults.

**Fig. 3:**
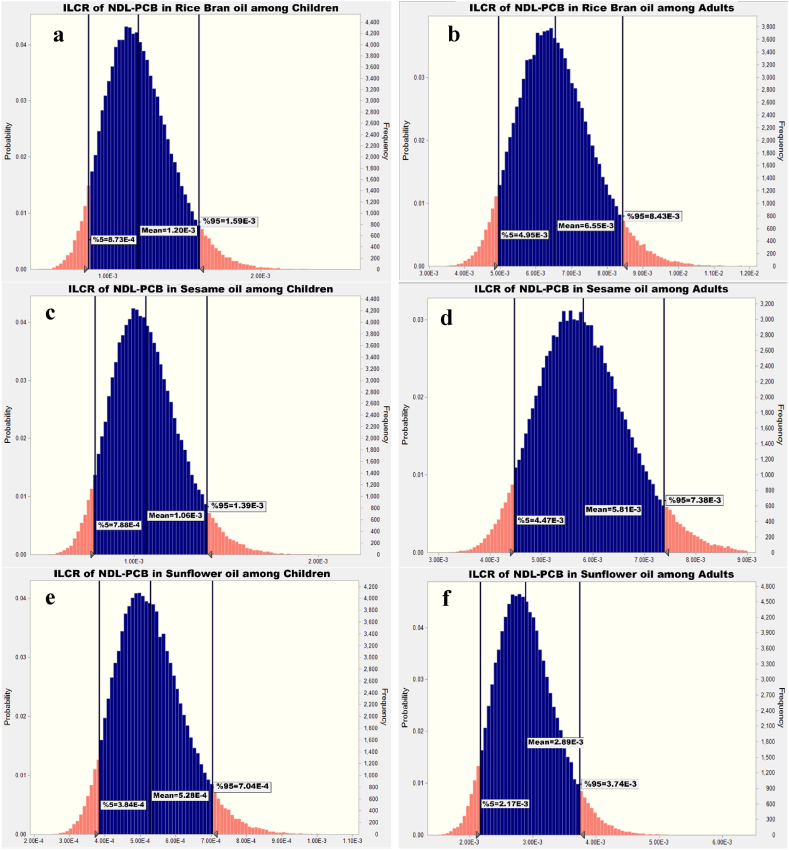
ILCR assessment of NDL-PCB in rice bran oil (a,b), sesame oil (c,d) and sunflower oil (e,f) among children and adults.

**Fig. 4:**
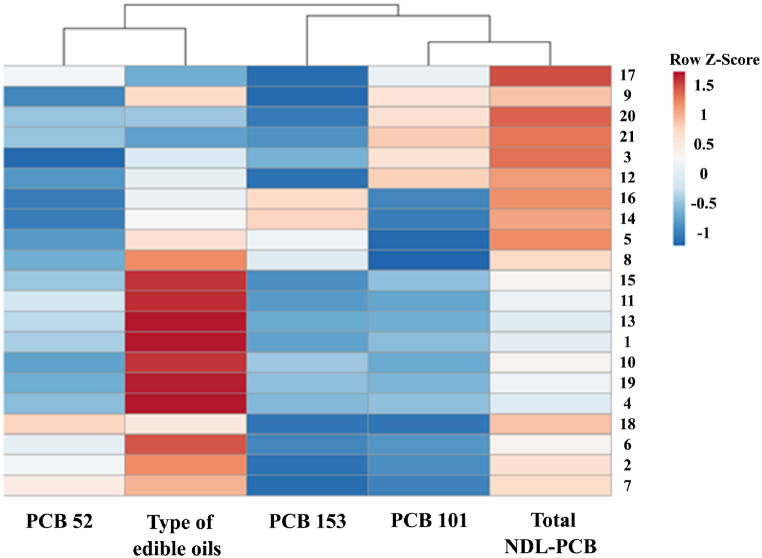
Heat map of 6 NDL-PCBs in edible oils samples.

Fig. 1, Fig. 2, Fig. 3, Fig. 4 indicate the dietary exposure to NDL-PCBs through the intake of various edible oils among both children and adults. The current work employed the Monte Carlo methodology to simulate the exposure to NDL-PCBs.

As shown in Figs. 1 and 95th percentile of exposure to NDL-PCB via animal oil were 7.80E-3 and 4.12E-2 among children and adults, respectively. Fig. 1 (a) shown that further investigation about NDL-PCB concentration in animal oil was required before applying any action in children group. Also, Fig. 1 (b) presented that the 95th percentile of simulated ILCR of expose to NDL-PCB via animal oils among adults is higher than children and equal to 4.12E-2, because the oil intake rate and exposure duration among adults are more than the children, if we want action conservative future investigation is needed and the ILCR value denotes there is high risk for cancer based on New York State Department of Health (NYSDH) [43].

Fig. 2(a–f) shown the probability of cancer due to expose to NDL-PCB via corn oil, frying oil and olive oil among children and adults. As Fig. 2(a) and (b) shown that 95th percentile of exposure to NDL-PCB via corn oil were 5.37E-4 and 3.04E-3 among children and adults, respectively. Fig. 2 (a) presented that further investigation about NDL-PCB level in corn oil was required before applying any action in children group that have used this edible oil. Also, Fig. 2 (b) displayed that the 95th percentile of ILCR due expose to NDL-PCB via corn oils among adults is higher than children and equal to 4.12E-2, so, action conservative is needed future investigation and the ILCR value denotes there is high risk for cancer based on NYSDH [43].

The ILCR of expose to NDL-PCB via frying oil among children and adults are presented in Fig. 2 (c) and 2 (d), respectively. This Figs shown that 95th centile of simulated ILCR among adults and children were 3.09E-3, and 5.37E-4, respectively; so, in both age group further investigation to evaluate the NDL-PCB concentration in frying oil were required to apply a suitable action.

In olive oil the 95th percentile of simulated ILCR among children and adults were more than this statistics in corn oil and frying oil; as observable in Fig. 2(e) and (f) the 95th percentile among adults and children were 1.06E-2, and 2.00E-3 respectively; so based on NYSDH guideline the risk level among children and adults were medium risk and high risk, respectively [43].

The 95th percentile of exposure to NDL-PCB via rice bran oil, sesame oil and sunflower oil among children group were 1.59E-3, 13.9E-3 and 7.04E-4 respectively (Fig. 3 (a), 3 (c) and 3 (e); which based on NYSDH for rice bran oil, sesame oil, sunflower oil the risk classification were high risk, medium risk and medium risk, respectively [43]. Also, as shown in Fig. 3 (b), 3(d) and 3(f) for adults group that expose to rice bran oil, sesame oil and sunflower oil the 95th percentile of simulated ILCR were 8.43E-3, 7.38E-3 and 3.74E-3 respectively. So, before any action based on the concentration of NDL-PCB via bran oil, sesame oil and sunflower oil is required.

### 3.2 Heat map analysis

The interactive heat map showed how clustering algorithms grouped data. Fig. 4 shows a cluster dendrogram to compare NDL-PCB congeners in samples. Sample correlation increases as Euclidean distance decreases. All samples are clustered into two to determine the association between three PCB types and amounts in edible oils. As the first category in different edible oils, PCB 153, PCB 101, and total PCBs show a relatively comparable pattern across all samples. While the other samples show a substantial link between PCB 28, PCB 153, and PCB 180, the second group includes food oils such sunflower, corn, olive, sesame, rice bran, frying, and animal oils. Levels of NDL-PCBs in samples can help differentiate the seven categories of edible oils. The heat map clusters columns with comparable PCB congeners that are highly correlated and shows the matrix's greatest and smallest congeners.

## 4 Conclusion

This study assessed PCB levels in Tehran-sold edible oil using modified QuEChERS extraction and GC-MS. MCS was used to estimate PCB cancer risk. In edible animal oils, PCB-101 had the greatest concentration, while PCB-153 in maize oils had the lowest. Most critically, the mean total NDL-PCBs in animal and vegetable oils was below the EU limit of 40 ng/g. Sunflower oil, corn oil, olive oil, sesame oil, rice bran oil, frying oil, and animal oil were examined using the heat map to determine contamination levels. Preliminarily visualizing the distribution of NDL-PCB allowed us to recognize similarities and differences in the data. The multivariate analysis could help identify or assess the different distributions of contamination status. The risk evaluation showed that in all edible oils, the 95th percentile of simulated ILCR was more than 10–5, so it can be threatening to health among both ageing groups. Also, according to the results, the 95th percentile of ILCR among adults was higher than among children because the edible oil ingestion rate among adults was higher than among children. By considering these issues, it is recommended to consumers that they decrease their use of edible oil or use edible oil that has a lower PCBs level. The limitations of present study include a low sample size and the non-availability of more PCB standards for analysis. Also, the strengths of our study were: I) variety of edible oils (vegetable and animal oils) analysis to detect PCB content; and II) detection of PCB levels carried out by GC-MS.

## Data availability

Not applicable.

## CRediT authorship contribution statement

**Hadi Eghbaljoo:** Writing – review & editing, Writing – original draft, Investigation. **Mohammad Rezvani Ghalhari:** Writing – review & editing, Writing – original draft, Software, Methodology, Formal analysis, Data curation. **Nabi Shariatifar:** Writing – original draft, Supervision, Project administration, Funding acquisition, Conceptualization. **Gholamreza Jahed Khaniki:** Validation, Resources, Data curation. **Ebrahim Molaee Aghaee:** Visualization, Validation, Resources, Conceptualization. **Mahmood Alizadeh Sani:** Writing – review & editing, Writing – original draft, Methodology, Investigation. **Elahe Mansouri:** Visualization, Investigation, Data curation. **Majid Arabameri:** Visualization, Software, Data curation.

## Declaration of competing interest

The authors declare that they have no known competing financial interests or personal relationships that could have appeared to influence the work reported in this paper.
